# RETRACTED: Alhakamy et al. Optimized Icariin Phytosomes Exhibit Enhanced Cytotoxicity and Apoptosis-Inducing Activities in Ovarian Cancer Cells. *Pharmaceutics* 2020, *12*, 346

**DOI:** 10.3390/pharmaceutics16020194

**Published:** 2024-01-30

**Authors:** Nabil A. Alhakamy, Usama A. Fahmy, Shaimaa M. Badr-Eldin, Osama A. A. Ahmed, Hani Z. Asfour, Hibah M. Aldawsari, Mardi M. Algandaby, Basma G. Eid, Ashraf B. Abdel-Naim, Zuhier A. Awan, Nabil K. Alruwaili, Amir I. Mohamed

**Affiliations:** 1 Department of Pharmaceutics, Faculty of Pharmacy, King Abdulaziz University, Jeddah 21589, Saudi Arabia; nalhakamy@kau.edu.sa (N.A.A.); smbali@kau.edu.sa (S.M.B.-E.); oaahmed@kau.edu.sa (O.A.A.A.); haldosari@kau.edu.sa (H.M.A.); 2 Center of Excellence for Drug Research and Pharmaceutical Industries, King Abdulaziz University, Jeddah 21589, Saudi Arabia; 3 King Fahd Medical Research Center, King Abdulaziz University, Jeddah 21589, Saudi Arabia; 4 Advanced Drug Delivery Research Group, Faculty of Pharmacy, King Abdulaziz University, Jeddah 21589, Saudi Arabia; 5 Department of Pharmaceutics and Industrial Pharmacy, Faculty of Pharmacy, Cairo University, Cairo 11562, Egypt; 6 Department of Medical Microbiology and Parasitology, Faculty of Medicine, King Abdulaziz University, Jeddah 21589, Saudi Arabia; hasfour@kau.edu.sa; 7 Department of Biological Sciences, Faculty of Science, King Abdulaziz University, Jeddah 21579, Saudi Arabia; malgandaby@kau.edu.sa; 8 Department of Pharmacology and Toxicology, Faculty of Pharmacy, King Abdulaziz University, Jeddah 21589, Saudi Arabia; beid@kau.edu.sa (B.G.E.); aaabdulalrahman1@kau.edu.sa (A.B.A.-N.); 9 Department of Clinical Biochemistry, Faculty of Medicine, King Abdulaziz University, Jeddah 21589, Saudi Arabia; zawan@kau.edu.sa; 10 Department of Pharmaceutics, Faculty of Pharmacy, Jouf University, Skaka 2014, Saudi Arabia; nabilalruwaili@gmail.com; 11 Department of Pharmaceutics and Industrial Pharmacy, Military Medical Academy, Cairo 11757, Egypt; miroami@gmail.com

The journal retracts the article, “Optimized Icariin Phytosomes Exhibit Enhanced Cytotoxicity and Apoptosis-Inducing Activities in Ovarian Cancer Cells” [1] cited above.

Following publication, concerns were brought to the attention of the publisher regarding duplicated images across other publications [2,3,4,5,6,7], representing different experimental conditions.

Adhering to our complaint procedure, an investigation was conducted that confirmed that Figure S2 of [1] is a duplicate of Figure 8 of [2], Figure 7 of [3], Figure 7 of [4], Figure 7 of [5], Figure 7 of [6] and Figure 4 of [7].

While the authors fully cooperated with the Editorial Office during the investigation, they were unable to satisfactorily explain the overlap of figures presenting different experimental conditions and meet the required quality standards of original images in order to consider a correction as per the journal’s original image requirements policy (https://www.mdpi.com/journal/pharmaceutics/instruc-tions#oriimages). As a result, the Editorial Board and Editor-in-Chief were unable to confirm the reliability of the findings and subsequently decided to retract this paper as per MDPI’s retraction policy (https://www.mdpi.com/ethics#_bookmark30).

This retraction was approved by the Editor-in-Chief of the journal *Pharmaceutics*.

The authors did not comment on this retraction.
